# From Targeted Needs Assessment to Course Ready for Implementation—A Model for Curriculum Development and the Course Results

**DOI:** 10.3390/ijerph20032529

**Published:** 2023-01-31

**Authors:** Rie Raffing, Sanne Larsen, Lars Konge, Hanne Tønnesen

**Affiliations:** 1 WHO Collaborating Centre, Clinical Health Promotion Centre, The Parker Institute, Bispebjerg & Frederiksberg Hospital, University of Copenhagen, DK-2400 Copenhagen, Denmark; 2 Centre for Internationalisation and Parallel Language Use, Faculty of Humanities, University of Copenhagen, DK-2300 Copenhagen, Denmark; 3 Copenhagen Academy for Medical Education and Simulation (CAMES), Centre for HR and Education, The Capital Region of Denmark, DK-2100 Copenhagen, Denmark; 4 WHO Collaborating Centre, Clinical Health Promotion Centre, Health Sciences, Lund University, SE-20502 Malmo, Sweden

**Keywords:** curriculum development, medical education, targeted needs assessment, scientific article, writing, research course, model

## Abstract

This article contributes guidance on how to approach the development of a course curriculum in general and presents a specific example from medical research education. The purpose of this study is to discuss a model for translating needs assessments of targeted learners into a course curriculum. The model employs established methods for data collection, such as different interview approaches and surveys. The authors argue that there is value in going from exploratory in-depth qualitative data collection methods to more conclusive rigorous quantitative methods when developing a course curriculum. In this way, the model is especially sensitive to the needs of targeted learners in the initial phase and at the same time offers a systematic and practical approach to curriculum development. The model is presented step-by-step with the aid of an empirical example of how to assess the needs of medical doctors in the publication process and develop an introductory course in writing an initial manuscript for publication. The article concludes that the proposed model gives curriculum developers a unique opportunity to explore the needs of targeted learners in depth, while systematically aiming towards conclusive decisions on curriculum content ready for implementation.

## 1. Introduction

Course curricula in medical education are continuously being developed and improved to comply with the increasingly complex health care environments in which medical students are trained to navigate. David Kern defines the curriculum as a planned educational experience that includes educational elements ranging from one session to an entire training program [1]. It is essential that a curriculum is properly developed because it is the foundation for learners and assessments in the course [2]. If a medical curriculum does not consider its learners, it risks including objectives that the learners are already familiar with—thus wasting the learners’ and teachers’ time—or omitting objectives that the learners need to know and thereby, as the ultimate consequence, risking patients’ health. A needs assessment of the targeted learners should therefore be an integrated part of course development [3].

In medical education, there are many wishes about the content and structure of education, for instance from simulation training. However, the development of proper curricula often skips the needs assessment and definition of goals and objectives and goes straight to defining educational strategies. Once the training curricula have been developed, another often-seen problem is that they are poorly implemented [4], although examples exist of implementation models tested in real life with promising results [5]. Thus, a plan for curriculum implementation should be an essential component when developing course curricula in medical education.

Kern’s approach to curriculum development for medical education includes six steps which may be taken in a convenient order, depending on the educational context, and which include different groups (Table 1).

In relation to Kern’s step 1, in an assessment of supervisors’ competence needs, we previously identified a problem in that some find it more difficult to support medical PhD students in writing their first article than students from other disciplines [6]. This led us to assess the needs of the targeted learners, i.e. medical doctors writing their first scientific manuscript for publication, thus related to Kern’s step 2 [7].

In the present study, we were inspired by Kern to develop a course by the practical operationalization of the other steps of his model for curriculum development. The aim was to develop and pilot test a model to translate the needs of medical PhD students into a related course curriculum, exemplified by a case from medical research education, and evaluate the results.

Below, we present the developed model in the materials and methods section, as well as the results of the model’s pilot test and the first implemented course in the results section.

## 2. Materials and Methods

Our approach to developing the model was to first use an inductive and explorative approach using interviews with open-ended questions to answer the question “What are the needs of the targeted learners?” and then to move to a more deductive approach using surveys to answer the question “Were the needs of the targeted learners met?”. 

### 2.1. Data Collection and Setting

In-depth interviews were chosen to use an explorative method, which is characterized by limiting preconceptions and predefined categories of the researcher [8]. Thereby, we expected to come as close as possible to an understanding of the immediate needs of the targeted learners. The purposes of the in-depth interviews were to investigate (a) how PhD students with a medical background experienced writing their first scientific article and (b) their views on whether a tailor-made course could help them overcome possible barriers [7].

Group interviews were chosen to gain insight into how the course form and content was interpreted by a group of targeted learners [9]. The purposes of group interview I were to explore (a) if the learning objectives and the course program were relevant and immediately comprehensible to PhD students with a medical background and (b) if the program corresponded to the learning objectives. The purposes of group interview II were to explore (a) if the overall purpose of the course had been fulfilled and (b) if the students’ expectations of the pilot course corresponded with the course description and program. Semi-structured interview guides were developed by a qualitative interview technique expert and an information specialist.

Direct observation, a method suggested by Kern, of the pilot course “Introduction to Scientific Article Writing” was chosen to directly observe how the course worked and was experienced by the participants [1]. 

A survey was chosen to complement the open-ended interview questions with a more closed-ended, structured, standardized, and less time-consuming method that would be a useful evaluation tool in the implemented course (Supplementary Materials). The purpose of the survey in the study was to obtain the students’ assessment of the overall aspects of the course, including content, planning, materials, and level of difficulty of their own efforts and of the lecturers’ ability to communicate, to involve the participants, and of the lecturers’ subject knowledge. The survey was identical to surveys used to evaluate similar courses.

The locations of the in-depth interviews, the group interviews, and the pilot course were in local homes, students’ offices, or at Frederiksberg Hospital, Copenhagen, Denmark.

### 2.2. Procedure

We approached the course development stepwise, inspired by Kern [1] and previously published the first two steps [6,7]. Step 3 of Kern’s model is about setting goals and objectives for the curriculum. In our model, this step runs parallel with a continued needs assessment. We developed goals and objectives based on in-depth interviews with individual learners and then we continuously refined the curriculum by involving the targeted learners through group interviews, a pilot test of the course, and finally an evaluation survey. Kern argues that goals and objectives serve the important functions of directing the choice of curricular content and assignment of relative priorities, as well as suggesting what learning methods will be effective [1]. We found it useful to let the development of goals and objectives take place in a process of interaction with the targeted learners. 

**Figure 1:**
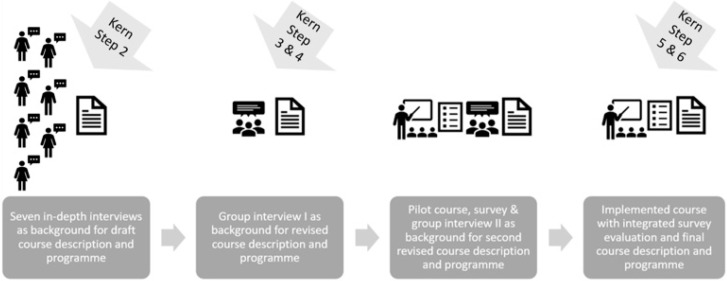
How targeted learners contributed to curriculum development.

In our case, steps 3–6 were thus initiated with a group interview regarding the course title, learning objectives, and course program, followed by a pilot course with a subsequent survey and a new group interview. Finally, we implemented the course with a related survey for evaluation. The inclusion of targeted learners in the development process formed the model for practical curriculum development (Figure 1). The implemented course has, since 2019, been a part of the PhD course catalogue at the Faculty of Health and Medical Sciences, University of Copenhagen, Denmark.

### 2.3. Participants

All participants were targeted learners for the curriculum under development. The recruitment of participants for the in-depth interviews was described elsewhere [7]. We used purposeful sampling to select the participants, which meant that they were included if they (a) were trained medical doctors, (b) had experience with writing their first scientific article, and (c) were active in research education as PhD students or enrolled in a specific curriculum as research students with plans to become PhD students within the following year. We sought to cover a wide range of medical research areas when recruiting participants. Participants for the first group interview and for the pilot course were recruited through local hospital networks and snowball sampling and participants for the second group interview were recruited on the pilot course. 

### 2.4. Data Analyses

All interviews were recorded digitally and transcribed verbatim. The in-depth interviews were coded using NVivo Qualitative Data Analysis Software, QSR International Pty Ltd., Melbourne, Australia. Kvale and Brinkmann suggest the following approach in their introduction to interview analyses with a focus on meaning: transcribe the interview; apply codes to the transcription; categorize the codes in meaning condensations; apply a theme which dominates the category; and lastly, expand on what is said to interpret meaning by developing structures and meaning relations that are not immediate in the transcribed text [10]. Our analysis of the in-depth interviews followed this approach inductively by applying specific codes to the interview transcriptions and applying themes to the developed categories. Our analyses of the group interviews followed the above-mentioned approach deductively by applying a thematic framework derived from the purposes of the interviews to the interview transcriptions. Thus, the analyses of the group interviews were steered by a primary focus on the target groups’ needs and requests to the course title, learning objectives, and program, as well as a secondary focus on interpretation. Surveys were employed after the pilot course and subsequently the median score and range were calculated. Open answers were analyzed with meaning condensation.

### 2.5. Ethical Considerations

All interviewees participated after informed consent was provided both orally and in writing. They were informed that they could withdraw from the study without consequences at any time. When the participants signed up for the pilot course, they were informed that the course was free of charge, that it was part of a project, and that data from the course content and evaluation would be used for research purposes. They were also given the opportunity to be informed about the results of the study when finalized. The project followed the GDPR and the Data Protection Law in Denmark. The Ethical Committee Board in Denmark does not consider interview studies and was therefore not consulted [11].

## 3. Results

The study included 16 participants (13 women and 3 men), representing a wide range of medical research areas (Table 2). Seven participated in in-depth interviews, four in group interview 1, seven in the pilot course, and three in group interview 2.

Stakeholders were also involved throughout the project. A representative from the PhD School at the Medical Faculty, which would later administer and offer the course, took part in planning and development meetings, and two teachers who lectured at the pilot course evaluated the course planning and content, as well as the preparation, participation, and knowledge level of the course participants.

The way Kern’s 6-step model was operationalized in this study is illustrated below (Table 3) and unfolded in the subsequent paragraphs.

### 3.1. In-Depth Interviews

For the previously reported in-depth interviews, the analysis resulted in four overall themes including several subthemes to the first two themes: (1) barriers to get started, (1a) difficulties writing, (1b) difficulties writing in English, (1c) difficulties with content, structure, and presentation, and (1d) difficulties navigating in the author group; (2) facilitators when getting started: participants’ tips, (2a) how to get started, (2b) how to write and (2c) how to write in English; (3) the role of the supervisor; and (4) course content [7].

### 3.2. Group Interview I

For the first group interview, the thematic framework consisted of themes of the relevance and comprehensibility of the learning objectives and course program, as well as correspondence between the course program and learning objectives. In group interview I, participants agreed that the content of the program and four out of six learning objectives were relevant and immediately comprehensible. One learning objective they understood differently than intended and one they were uncertain of the meaning. 

They would like the course program to be communicated clearer and with more specific content and a revised title. “Well, when you say it is about the writing process it makes a hundred percent more sense, because that is where you think: ‘I am standing in front of a mountain!’” (Participant 9). “Yes, I have noted that this should be communicated much clearer: Writing process” (Interviewer). 

The participants wanted individual writing time with their own article, seeing progression from day to day and to obtain a product for each day. They did not prefer group work but asked for tips and tricks, templates, guidelines, and a target to aim for. “My point is, make a target—go for the target. We know how to do that, right?” (Participant 9). “Yes exactly! Don’t look in another direction, just focus and get going” (Participant 8).

### 3.3. Pilot Course: Surveys and Group Interview II

In the survey of the pilot course, the median participant satisfaction was 6 [5,7] on a Likert scale from 1–7. All the students found that the course provided them with knowledge that would be concretely useful in the future. The thematic analyses of open text responses from the evaluation showed that the participants overall found it was a great and useful course, that the selection of lecturers was good, that they wanted even more time and teaching on academic English, and that some of the lectures overlapped in focus.

Prior to this course, full in-depth interviews with 20 PhD supervisors showed medical PhD students required more supervision when writing their first article [6]. In the survey of the pilot course, median teacher satisfaction was 6.5 [6,7] on a Likert scale from 1–7. One teacher commented on the importance of evidence-based teaching, and another commented on increasing educational strategies and time for academic English in the course.

For the second group interview the thematic framework consisted of the fulfilment of the purpose of the course and correspondence between course description and program. In group interview II, participants found the course appropriate for PhD students, as well as research students, and would all have chosen it if it was offered through the course catalogue of the PhD School.

All agreed that the overall purpose of the course had been fulfilled and that the course description and program corresponded with their experience of the course. ”It has been very rewarding because we have been presented with much to think about for the future writing process, both on day one, two and three” (Participant 14). “I also think it was good that it was hands-on and that you could bring your own article” (Participant 16). 

They pointed out areas that had worked especially well and areas of improvement. Most suggestions to improvement were minor issues, such as more time for some subjects, overlapping lectures, and more specific instructions for homework. ”I would like to have had even more time for academic English because I think it is the most difficult“(Participant 14). “I agree that it is a repetition of the same subject, it has just been communicated differently” (Participant 14). “The homework should probably have been introduced better and we should have sent it to you before the second course day” (Participant 16). 

The participants found the course inspiring and expressed that it provided them with the tools to start the writing process. ”I think it was nice to get some tools” (Participant 15). “I agree, it was nice […] I learned a lot, that I did not know before” (Participant 16). “It is so specific, and we can apply it directly to what we are working with” (Participant 15). “It has been so inspiring so now I am ready!” (Participant 16) [other participants are nodding]. 

### 3.4. Subsequent Adjustments

A lecture on the editor’s perspective was taken out to avoid overlapping and individual writing time was increased by 90 min, involving two supervisors with specific focus on language to accommodate the need for more time on academic English.

### 3.5. Implemented Course: Survey

In the survey of the implemented course (spring 2019) (Table 4), the median participant satisfaction was 6 [5,7] on a Likert scale from 1–7. All the students found that the course provided them with knowledge that would be concretely useful in the future. The thematic analyses of open text responses from the evaluation showed that the participants appreciated the selection of lecturers and the teaching on how to structure an introduction. They still requested more teaching on writing in English. 

## 4. Discussion

This study showed that initiating course development with an explorative method provided rich and relevant data for drafting a course description and program. To assess this draft in a group interview confirmed the initial solid knowledge base from the in-depth interviews [7] and revealed important points of improvement. Pilot testing the course showed that it was possible to carry out in practice, and the subsequent survey and group evaluations showed that minor adjustments could improve the course further before implementation. Evaluation was integrated in the end of the final course program to secure continuous improvement. 

We propose that our operationalization of Kern’s model is sensitive to the needs of targeted learners and at the same time offers a systematic and practical approach to curriculum development which translates the needs of targeted learners in a clinical context into a course curriculum ready for implementation.

### 4.1. Application of Methods in Targeted Needs Assessment and Curricular Development

There are good examples of how a single method has been used in a targeted needs assessment to successfully guide the development of medical curricula. Peluso et al. used structured interviews to guide the development of a structured internal medicine curriculum for medical interns in Botswana [12], and Lamb et al. used a 25-item survey for the development of a surgical sub-internship curriculum in the USA [13]. These studies included a single method to assess the needs of their targeted learners, but as we are putting on the agenda with this study, it is worth reflecting on if a single method is enough, as well as how to decide which method(s) to use in a targeted needs assessment followed by curriculum development.

Other studies on course development have also reported the advantages of initiating with an explorative approach, followed by a less explorative one. In-depth interviews with seven experts were followed up by a survey when developing a clubfoot treatment curriculum in Africa [14], and in-depth interviews were followed up by focus groups when developing a suicide prevention program and a health promotion program in Iran [15,16]. A focus group informed the development of a survey to aid curriculum development on chronic pelvic pain in USA [17].

These studies also moved from explorative methods to more quantitative methods when assessing the needs of targeted learners. However, it was not within their scope to explicitly reflect on the order of the methods employed. Smythe et al., developing clubfoot treatment, concluded that using both quantitative and qualitative methods was essential for course development of training courses [14] but, apart from this study, the methodological composition of needs assessments was not commented on in the studies. This could be because the focus of the studies was more on the product of curriculum development than on the process.

### 4.2. Interpretation of Needs Assessment in a Broader Context

Lynore Desilets advised that in the interpretation of needs assessment data, a clear distinction should be made between lack-of-knowledge needs and attendant or associated issues [18]. In our study, the role of the supervisor, as well as insecurity when writing, fell into the category of associated issues. Because they were more of a psychological character and fell out of the scope of creating a practical writing course, we omitted them when selecting the major components of the curriculum. 

However, recognizing the important role of the supervisor and the students’ explicit need of close and constructive supervision of their first article, we sought to initiate a positive dialogue between student and supervisor by including homework where the students should ask the supervisor to provide a good article and a comment on why the supervisor found the article good. 

Moreover, we recognized that the struggles of writing for some participants seemed counterproductive and that they seemed limited by insecurities in their role as the main author and in how their products were received by their supervisors and others. This led us to include elements of writing processes in the curriculum, teaching the participants that writing takes lots of practice, and that insecurity and finding it difficult to write is okay and normal. We wanted to prepare the novice writers to recognize the challenge of writing as familiar and, for some, even as a welcome challenge.

### 4.3. Bias, Limitations and Strengths

We described earlier the possible selection bias of including more women than men in the in-depth interviews and argued that this gender distribution seems acceptable because most PhD students within healthcare in Denmark are women [7]. 

The interpretations of the findings of both the in-depth and group interviews were discussed by the two interviewers to minimize the risk of confirmation bias.

The sample size of seven participants in the in-depth interviews and four and three participants in the two group interviews might be a limitation. However, in the in-depth interviews, participants were included until saturation, which occurred after seven interviews. Four and three participants are small sample sizes even for group interviews, where five to eight participants are recommended [19]. More participants were invited to the interviews but cancelled last minute due to illness and acute clinical work, which are issues that are hard to avoid in a real-life setting.

Another limitation of this study is that the model was only tested among medical doctors from the Capital Region of Denmark studying for a PhD degree. Therefore, the results are not immediately generalizable to the PhD student population. It is recommended to test the model within other disciplines and in other geographical settings for generalizability and application to practice.

Another limitation is that the evaluation only assessed the students’ subjective experience of the course, and the students might not have a full overview of their needs. However, this study was also supported by one of our previous studies among PhD supervisors, who argued that PhD students with a medical background have more supervision needs than other PhD students when writing their first manuscript [6]. We do recommend a more comprehensive evaluation model that also takes the students’ motivation, the learning effect, and long-term academic achievement into account. Outcomes of the evaluation model should be measured by validated scales for motivation, context-specific tools, such as multiple-choice questionnaires for learning effect, and publicly available research profiles for academic achievements. 

The strengths of this study are that it includes a variety of methods and that the model was tested in real life with targeted learners.

### 4.4. Perspectives

The developed model was tested on participants with a clinical background, but due to its solid methodological foundation and general appeal, it is very likely to be useful in any kind of curriculum development. It would be useful to test the model in other geographical or educational settings. Moreover, the model was tested in an onsite learning environment. It would be useful to test it in an online or flipped learning environment as well. 

## 5. Conclusions

The process of going from exploratory to conclusive methods has proven useful to involve the targeted learners. The model gives curriculum developers a unique opportunity to assess the needs of the targeted learners in depth, while steering systematically towards firm decisions on curriculum content ready for implementation. Therefore, we suggest this or a similar model to be used when translating the targeted needs into a course curriculum. 

## Figures and Tables

**Table 1:** David Kern’s stepwise approach to curriculum development [1].

Step	Description	Groups Included
1	Problem identification and general needs assessment	Consultants/experts
2	Targeted needs assessment	Targeted learners
3	Goals and objectives	Learners/teachers/curriculum developers
4	Educational strategies	Curriculum developers
5	Implementation	Curriculum developers Personnel Stakeholders
6	Feedback and evaluation	Users of curriculum Stakeholders

**Table 2:** Study participants.

Participant	Gender	Research Area	Participation
1 *	Man	Pediatrics	In-depth interview
2 *	Woman	Pediatrics	In-depth interview
3 *	Woman	Cancer	In-depth interview
4 *	Woman	Internal medicine	In-depth interview
5 *	Woman	Neurology	In-depth interview
6 *	Woman	Pediatrics	In-depth interview and Group interview 1
7 *	Woman	Diabetes	In-depth interview and Group interview 1
8	Woman	Rheumatology	Group interview 1
9	Man	Dermatology	Group interview 1
10	Woman	Radiology	Pilot course
11	Woman	Occupational and Environmental Medicine	Pilot course
12	Man	Endocrinology	Pilot course
13	Woman	Dermatology	Pilot course
14	Woman	Clinical Pharmacology	Pilot course and Group interview 2
15	Woman	Diabetes	Pilot course and Group interview 2
16	Woman	Gastroenterology	Pilot course and Group interview 2

* Participated in the in-depth interviews of the targeted needs assessment previously reported [7].

**Table 3:** The operationalization of Kern’s 6-step model in this study.

David Kern’s 6 Steps Including Groups			Operationalization in This Study		
Step	Description	Groups	Groups	Methods	Results
1	Problem identification and general needs assessment	Consultants/ experts	PhD supervisors	In-depth interviews	Medical PhD students require more supervision when writing their first research article [6].
2	Targeted needs assessment	Targeted learners	Medical PhD students	In-depth interviews	Medical PhD students are in need of, and have specific requests to, a course on initiating the research writing process [7].
3	Goals and Objectives	Learners/teachers/ Curriculum developers	Targeted learners	Group interview	- Revise title - Revise some learning objectives - Communicate program clearer
4	Educational Strategies	Curriculum developers	Targeted learners	Group interview	- Revise content: want a target to aim for, guidelines, and a daily product. - Revise educational methods: want individual writing time.
This study adds	Pilot course		Targeted learners	Direct observation, survey and group interview	- Median satisfaction 6 [5,7]. - All found the knowledge useful. - Many found the teaching very good. - Many wanted more teaching on writing in English and less overlapping lectures. - Purpose was fulfilled and course description and program were satisfying.
			Teachers	Survey	- Median satisfaction 6.5 [6,7]. - Evidence-based teaching is vital. - More educational strategies. - More time for academic English.
5	Implementation	Curriculum developers Personnel Stakeholders	Teachers Stakeholders	Course management meeting	PhD course, “Getting started: Writing your first manuscript for publication”, Faculty of Health and Medical Sciences, University of Copenhagen, Denmark.
6	Feedback and Evaluation	Users of curriculum Stakeholders	Targeted Learners	Survey	- Median satisfaction 6 [5,7]. - All found the knowledge useful. - Many appreciated the selection of teachers and the teaching on introduction structure. - Many wanted more teaching on writing in English.

**Table 4:** The first implemented course title, learning objectives, and program.

Title Getting Started: Writing Your First Manuscript for Publication		
Learning objectives (1) To be able to define the purpose of your own article (2) To be able to select relevant content for your own article (3) To be able to structure the content of your own article (4) To be able to write the sections of your own article		
Programme Day 1: Purpose and Focus	Day 2: Content and Structure	Day 3: Writing
Preparations for day 1 (app. 2 days) Background literature Overview of the results Read author instructions of journal Supervisor example of a good article Read articles and course material	Preparations for day 2 (app. 2 h) Describe your article in four sentences Describe what your article is about in one core sentence.	Preparations for day 3 (app. 3 days) Background section and article outline Consider individual writing supervision
Welcome and Introduction	Discussion on challenges from homework	Discussion on challenges from homework
Lecture: Examination of selected articles; Purpose, introduction, discussion	Lecture: Writing strategies and processes	Lecture: Academic English
Reflection and writing exercise: What works for you and what can you use from the lectures?	Reflection and writing exercise: What works for you as an author—why? What can you use from the lecture?	Supervised individual writing in groups divided according to the lectures above
Lecture: Purpose of your article Research Question (PICO)	Lecture: Content; What should you include in your article? Lecture: Structure; different types of medical research articles	Lecture: Know your journal Reference formats, peer-review, authorship, cover letters, instructions for authors etc.
Reflection and writing exercise: What can you use from the lectures in your own article?	Reflection exercise: The structure of your article	Completion and evaluation
Introduction to homework for day two	Introduction to homework for day three	*-*
Product of the day: Draft of the purpose of your article	Product of the day: Idea of content and draft outline of your article	Product of the day: Draft of an individually selected focus area in the article

## Data Availability

The data consist of interview transcriptions and sharing these data could compromise individual privacy. Therefore, data will only be shared upon reasonable request with the intention to collaborate with the data owners and under the understanding that the anonymity of the project participants will be honored.

## Supplementary Materials

The following supporting information can be downloaded at: https://www.mdpi.com/article/10.3390/ijerph20032529/s1.
