# Supplementary material for: From Targeted Needs Assessment to Course Ready for Implementation—A Model for Curriculum Development and the Course Results
Source: Int J Environ Res Public Health. 2023 Jan 31;20(3):2529. doi: 10.3390/ijerph20032529 (PMC9915190; doi:10.3390/ijerph20032529)
Supplement: Supplementary file 1 [file ijerph-20-02529-s001.zip › ijerph-2129928-supplementary.pdf]

Supplementary Materials

Evaluation of the course *Introduction to Scientific Article Writing*
Date

You have just participated in the course *Introduction to Scientific Article Writing* and we would like to ask your opinion of the course. Your answers are valuable for us, because they provide us with a greater knowledge of how we can improve the course. Therefore, we hope that you will fill in the following short survey which takes about 10 minutes to complete.

Yours sincerely,
The Course Coordinators

1. What do you think of the course in general? (Only one answer per question)

	Extremely unsatisfactory	Very unsatisfactory	Unsatisfactory	Neither satisfactory nor unsatisfactory	Satisfactory	Very satisfactory	Extremely satisfactory
Content	☐	☐	☐	☐	☐	☐	☐
Planning and execution	☐	☐	☐	☐	☐	☐	☐
Relevance of course materials	☐	☐	☐	☐	☐	☐	☐
Level of difficulty	☐	☐	☐	☐	☐	☐	☐

2. How will you assess your own contribution to the course? (Only one answer per question)

	Extremely unsatisfactory	Very unsatisfactory	Unsatisfactory	Neither satisfactory nor unsatisfactory	Satisfactory	Very satisfactory	Extremely satisfactory
	☐	☐	☐	☐	☐	☐	☐

3. How will you assess the lecturers on the course? (Only one answer per question)

3a Ability of the teacher to communicate the material in a vibrant and interesting way

	Extremely unsatisfactory	Very unsatisfactory	Unsatisfactory	Neither satisfactory nor unsatisfactory	Satisfactory	Very satisfactory	Extremely satisfactory
Name of lecturer 1	☐	☐	☐	☐	☐	☐	☐
Name of lecturer 2	☐	☐	☐	☐	☐	☐	☐
Etc.	☐	☐	☐	☐	☐	☐	☐

3b Ability of the lecturer to involve the course participants in the lessons

	Extremely unsatisfactory	Very unsatisfactory	Unsatisfactory	Neither satisfactory nor unsatisfactory	Satisfactory	Very satisfactory	Extremely satisfactory
Name of lecturer 1	☐	☐	☐	☐	☐	☐	☐
Name of lecturer 2	☐	☐	☐	☐	☐	☐	☐
Etc.	☐	☐	☐	☐	☐	☐	☐

3c Subject knowledge of the teacher

	Extremely unsatisfactory	Very unsatisfactory	Unsatisfactory	Neither satisfactory nor unsatisfactory	Satisfactory	Very satisfactory	Extremely satisfactory
Name of lecturer 1	☐	☐	☐	☐	☐	☐	☐
Name of lecturer 2	☐	☐	☐	☐	☐	☐	☐
Etc.	☐	☐	☐	☐	☐	☐	☐

4. What would you like more of on the course?
5. What would you like less of on the course?
6. Did the course provide you with knowledge that you can use specifically in your further training? (Only one answer)

☐ Yes ☐ No ☐ Do not know

7. Do you have other comments, that you would like to share with the course management?

Thank you!
